# Investigation of Zirconium Effect on the Corrosion Resistance of Aluminum Alloy Using Electrochemical Methods and Numerical Simulation in an Acidified Synthetic Sea Salt Solution

**DOI:** 10.3390/ma11101982

**Published:** 2018-10-15

**Authors:** Yong-Sang Kim, Jong Gil Park, Byeong-Seon An, Young Hee Lee, Cheol-Woong Yang, Jung-Gu Kim

**Affiliations:** 1 School of Advanced Materials Science and Engineering, Sungkyunkwan University, 2066, Seobu-Ro, Jangan-Gu, Suwon, Gyeonggi-Do 16419, Korea; skybyego@gmail.com (Y.-S.K.); absabs22@skku.edu (B.-S.A.); cwyang@skku.edu (C.-W.Y.); 2 Department of Energy Science, Center for Integrated Nanostructure Physics, Institute for Basic Science, Sungkyunkwan University, 2066, Seobu-Ro, Jangan-Gu, Suwon, Gyeonggi-Do 16419, Korea; parkjg84@skku.edu (J.G.P.); leeyoung@skku.edu (Y.H.L.)

**Keywords:** aluminum alloy, corrosion, transmission electron microscopy, zirconium, intermetallic compound

## Abstract

Corrosion resistance of Zr that has been added to an Al alloy (U1070) is higher than that of a commercial Al alloy (A1070). A decreasing number and size of Al_3_Fe intermetallic particles (IMPs) were observed by electron microprobe analysis and transmission electron microscopy. Based on the numerical corrosion simulation, it was confirmed that decreasing the number and size of IMPs was favorable for improving the corrosion resistance of the Al alloy due to the reduction of the galvanic effect. In addition, Al_3_Zr was found to be insignificant in promoting galvanic corrosion within the Al matrix. Thus, Zr is an advantageous alloying element for improving the corrosion resistance of the Al alloy.

## 1. Introduction

Applications of aluminum (Al) alloys have expanded across numerous industries due to their desirable properties, including their light weight, high heat conduction, and favorable electrical and mechanical characteristics [1]. One typical applications for an Al alloy is as a heat exchanger in an air conditioner [2,3,4,5]; notably, Al is a commonly used material for heat exchange because of its high formability, good specific strength, low density, and high thermal conductivity [6,7]. In addition, it is a more economical material than copper, which is the original material that is used in heat exchanger applications. Despite the advantages of Al as a material for use in heat exchange, corrosion remains an issue. Generally, the corrosion of an Al heat exchanger is examined in a specific high-concentration salt solution such as acidified synthetic sea water, which is normally applied in the corrosion test of the automotive industry, because the operating environment of an Al heat exchanger is similar to that of a car. Thus, the improvement of Al corrosion resistance in this highly concentrated salt water is necessary for acceptance of the corrosion durability test and for increasing corrosion reliability.

The Al alloy is composed of multi-element, which influences the corrosion resistance of the Al alloy. The concept of microgalvanic corrosion between the Al matrix and the intermetallic particles (IMPs) is the most common corrosion mechanism for the Al alloy [8,9,10,11,12,13,14]. Formed IMPs in the Al alloy generally act as a cathodic site, because the alloying elements are more noble than Al, resulting in a galvanic reactions between IMPs and the Al matrix is occurring. Efforts to understand the microgalvanic reaction between IMPs and the Al matrix have been conducted by experimental and computational methods [15,16,17]. Although many scientific and engineering outputs have been suggested, further research is necessary on their elemental effects and geometric factors.

Thus, in order to improve the corrosion resistance of the Al alloy, the control and modification of IMPs in the Al alloy are important concepts for consideration. In the 1xxx series of Al alloy, the precipitated IMPs are almost related to the impurity elements. Especially, iron (Fe) is the most harmful impurity in Al, and is always present in alloys made from commercially pure base materials [18,19]. Since the maximum equilibrium solubility of iron in solid Al is very low (at 0.05 wt %), most of the Fe forms Fe-rich intermetallic compounds, which appear as needles or plates in the microstructure [20,21,22]. In the case of an Al alloy, the difference in corrosion potential between the Al–Fe IMP and the Al matrix is very high, creating a large driving force for microgalvanic corrosion. Although Fe removal methods have been employed, such as the precipitation and separation of Fe-rich phases from the liquid Al melt [23,24,25] and the addition of other elements (e.g., manganese, chromium) [26,27,28,29], the additives have a negative effect on the corrosion resistance of the Al alloy [30] and the application of this method has economical limitations.

In previous studies, the effect of zirconium (Zr) on the grain refinement in Al has been investigated [31,32], with the results indicating that grain refinement is attributed to the peritectic reaction induced by the properitectic Al_3_Zr phase. Although the grain refinement effect has been researched by various institutes and universities, the influence of the refinement after adding Zr to the corrosion resistance of Al has not yet been considered. Also, the effect of Zr on the distribution of the Al–Fe IMPs, which are the major cause of microgalvanic corrosion in the Al alloy, requires further research. Meanwhile, the corrosion potential of Al_3_Zr IMPs is very close to that of pure Al in high salt electrolyte conditions [32], indicating that the driving force for microgalvanic corrosion between Al_3_Zr and the Al matrix is very low. Based on both concepts (i.e., refinement and the small difference of corrosion potential), the influence of Zr on the corrosion resistance of the 1xxx Al alloy was investigated in the present study. For the analysis of corrosion in the tested 1xxx Al alloys, electrochemical tests [e.g., potentiodynamic polarization tests and electrochemical impedance spectroscopy (EIS)] and numerical simulations in various IMP distribution situations were conducted. In addition, the identification of Al–Fe and Al–Zr IMPs was verified by scanning electron microscopy (SEM), electron probe microanalysis (EPMA), and transmission electron microscopy (TEM).

## 2. Experimental Methods

### 2.1. Specimens and Solution Preparation

To evaluate the effects of Zr on IMPs in the Al matrix, two tubes consisting of Al alloys (A1070 and U1070) were prepared by casting and mechanical extrusion processes. Although Zr was added in U1070 to investigate the Zr effect, it had the same concentration of Fe, Si, and Cu as the A1070 commercial Al tube. In the casting process, pure Al ingot (99.9%) and three mother alloys (Al-20 wt % Fe alloy, Al-40 wt % silicon alloy, and Al-5 wt % Zr alloy) were charged into a graphite crucible at 710 °C. After all of the components were melted, the degassing process was carried out by nitrogen gas purging. Then, mechanical agitation was performed to uniformly mix all of the alloying elements. Molten metals were injected into the 20-cm diameter of a cylindrical mold and air-cooled. Subsequently, the mold was subjected to a homogenization treatment at 490 °C for 10 h. The chemical composition of both Al alloys was analyzed using ICP-MS with Agilent 7500 (Agilent Technologies, Santa Clara, CA, USA), as shown in Table 1. In addition, the specimens for the tests were generally achieved at the center of the cylindrical mold to avoid any surface defects and microstructure differences.

### 2.2. Electrochemical Tests

To evaluate the corrosion resistance of two Al alloys (i.e., A1070 and U1070), a potentiodynamic polarization test and EIS were conducted by use of multi-potentiostat/galvanostat instrument VSP-300s (Bio-Logic Science Instruments, Seyssinet-Pariset, France). A three-electrode cell was constructed with the Al alloys as the working electrode (WE), two pure graphite rods as the counter electrode (CE), and a saturated calomel electrode (SCE) as the reference electrode (RE). Prior to the electrochemical tests, the Al specimens were abraded with SiC paper with grit sizes from 220 to 600. The prepared tubes were then covered with silicone rubber, leaving an area of 0.09 cm^2^ unmasked. The prepared specimens were exposed to a salt water–acetic acid solution at 47 °C under the aerated condition, which is based on ASTM G85 [33]. Table 2 gives the chemical composition of the test solution. The specimens were rinsed ultrasonically with ethanol, and finally dried with nitrogen gas. Before all of the electrochemical tests, the specimens were immersed in a test solution for one hour to attain a stable state. Potentiodynamic polarization measurement was performed at a potential sweep of 1 mV/s from an initial potential of −250 mV versus an open circuit potential (OCP) to the final potential of 300 mV_SCE_. After polarization tests, the cross-section of both specimens was observed using an optical microscope. EIS tests were performed at an OCP in the test solution to investigate the surface properties. The frequency range of the EIS tests was from 100 kHz to 10 mHz, with an alternating current amplitude of ±10 mV. The impedance plots were interpreted on the basis of an equivalent circuit using a suitable fitting procedure by use of the ZsimpWin software (Princeton Applied Research, Oak Ridge, TN, USA). Each test was carried out in three replicates to ensure its reproducibility.

### 2.3. Analysis of IMPs

The distribution and composition of IMPs are important factors that are related to the corrosion resistance of the Al alloy. Thus, in order to analyze the IMPs in both Al alloys, various surface analyses were performed in this study. First, the distribution of the IMPs in the Al alloy (especially Al–Fe IMPs) was observed via field emission EPMA (FE-EPMA) in a JXA-5830F (JEOL, Tokyo, Japan). In addition, energy dispersion spectroscopy (EDS), and high-resolution TEM (HR-TEM) in JEM-ARM300F (JEOL, Tokyo, Japan) were performed for the detailed observation of the size, shape, and composition of the IMPs. Prior to FE-EPMA analysis, the specimen surfaces were polished with a 0.03-μm alumina (Al_2_O_3_) solution. Prior to HR-TEM analysis, the specimen was sliced via the machining and ion milling processes to yield thin specimens (10 μm) without thermal influence.

### 2.4. Modeling and Boundary Conditions

To evaluate the influence of IMPs in the Al matrix on the corrosion resistance of the Al alloy, numerical simulations were conducted on various IMP distribution conditions. Although the propagation of corrosion due to microgalvanic action between IMPs and the Al matrix is an important aspect in the corrosion resistance, the corrosion behavior at the transient surface state is focused in this study for the detailed analysis and verification of the electrochemical result, which only reflected the temporary corrosion resistance. Thus, the boundary element method (BEM) was used in this study.

**Figure 1:**
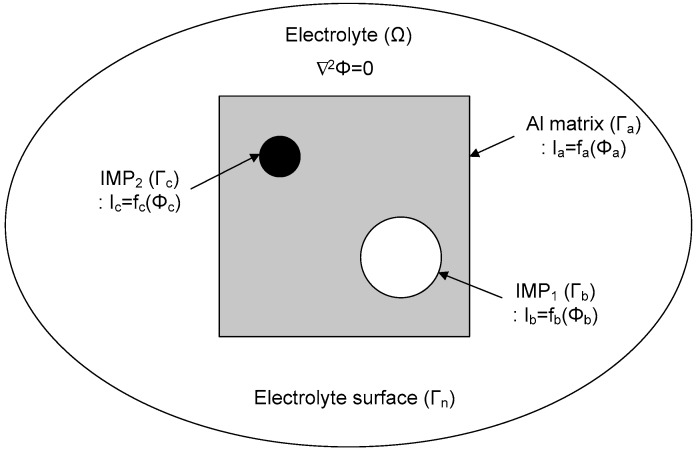
Boundary conditions for corrosion simulation of intermetallic particles (IMPs) in an aluminum matrix.

The schematic boundary condition for the corrosion simulation in this study is shown in Figure 1; the boundary condition has electrolyte domains (*Ω*), and is surrounded by the surface of the electrolyte *Γ*_n_, the surface of the Al matrix *Γ*_a_, and the surfaces of the IMPs *Γ*_b_ and *Γ*_c_. The electrolyte of conductivity (*σ*) is uniform over the whole domain, and there is no current loss. The potential field in the electrolyte domain can be modeled by use of the Laplace equation, as follows [34]:

(1) $$\nabla^{2}\Phi =0$$

Here, *Φ* is the electrical potential, which is the potential relative to a reference electrode.

The Laplace equation is solved using the following boundary conditions:

*i* = *i*_0_, on *Γ*_n_ (2)

*i*_a_ = *f*_a_(*Φ*_a_), on *Γ*_a_ (3)

*i*_b_ = *f*_b_(*Φ*_b_), on *Γ*_b_ (4)

*i*_c_ = *f*_c_(*Φ*_c_), on *Γ*_c_ (5)

Here, *Γ* is the entire surface of the electrolyte domain, which includes *Γ*_n_ (electrolyte surface), *Γ*_a_ (Al matrix), *Γ*_b_ (IMP_1_), and *Γ*_c_ (IMP_2_). Additionally, *f*_a_(*Φ*_a_), *f*_b_(*Φ*_b_), and *f*_c_(*Φ*_c_) are nonlinear functions on each surface that represent the polarization behavior of each part. Based on this, the potential and current over the whole surface can be calculated.

**Figure 2:**
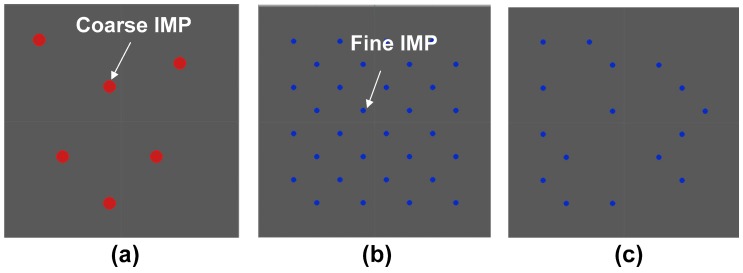
IMPs distributed models for corrosion simulation: (**a**) large size of IMPs in the Al matrix (large IMPs area); (**b**) small size of IMPs in the Al matrix (large IMPs area); and (**c**) small size of IMPs in Al matrix (small IMPs area).

IMP distribution models for corrosion simulation were created using the Rhinoceros 3D drawing software (McNeel, Seattle, WA, USA). Three cases were considered for the detailed interpretation of corrosion between the IMPs and the Al matrix. Figure 2 shows the modeling cases for the corrosion simulation in this study. To simplify the corrosion simulation, the model for IMPs was fixed to the circle, and the location of particles was set such that they are randomly distributed in the rectangular grid of the Al matrix. The size of the Al matrix was set to 2 mm × 2 mm. The polarization parameters of IMPs were based on the results of previous research [35], and are listed in Table 3. The only applied IMPs were Al_3_Fe and Al_3_Zr, because Al_3_Fe is a significant IMP for microgalvanic corrosion in 1xxx Al alloys, and the Zr effect is the main focus of this study. Generally, the anodic Tafel slope of Al in salt water is very low due to the fast anodic reaction, while the cathodic Tafel slope in an aerated environment is high due to the diffusion-limited current caused by oxygen diffusion. Thus, the Tafel slopes that were used for the corrosion simulation consider both the anodic and cathodic properties of Al. Detailed conditions for the corrosion simulation model are listed in Table 4.

## 3. Results and Discussion

### 3.1. Corrosion Behavior

**Figure 3:**
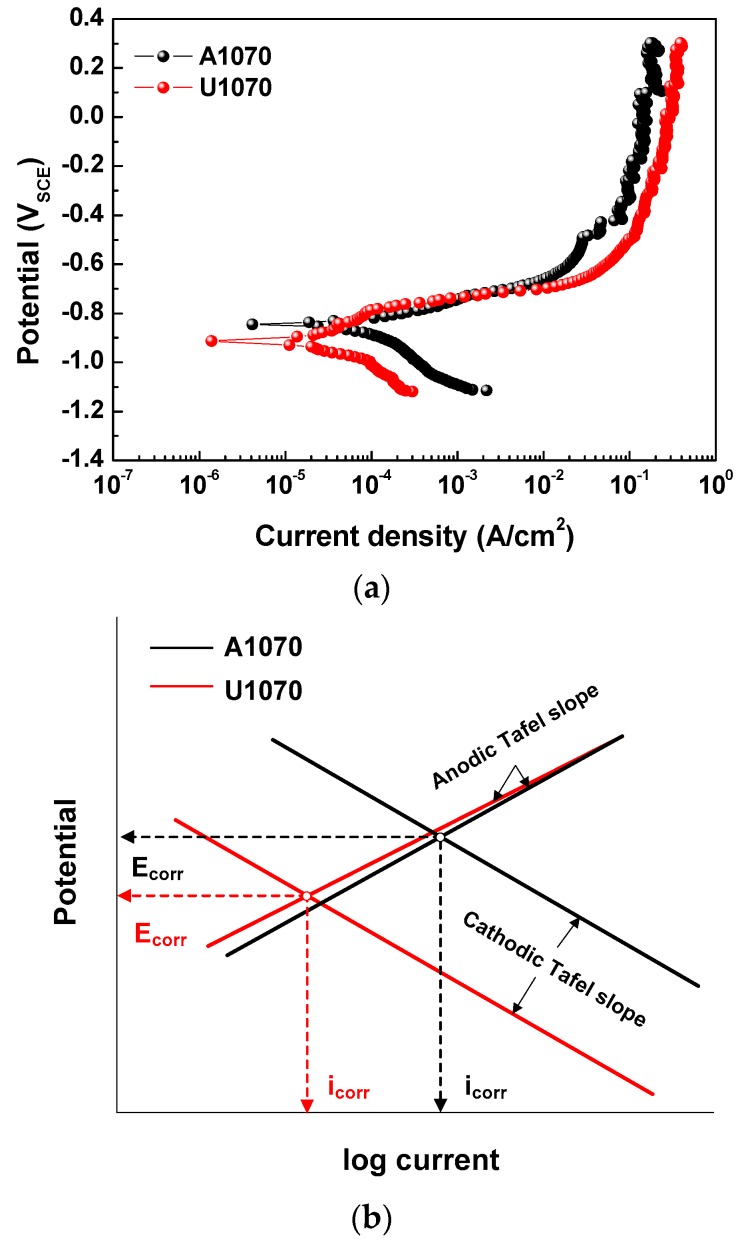
(**a**) Polarization curves and (**b**) schematic Tafel slope of A1070 and U 1070 in a salt water–acetic acid solution at 47 °C.

**Figure 4:**
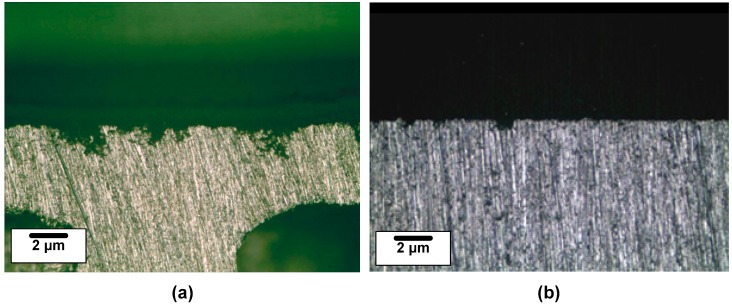
Cross-sectional images of (**a**) A1070 and (**b**) U1070 after the polarization tests.

**Figure 5:**
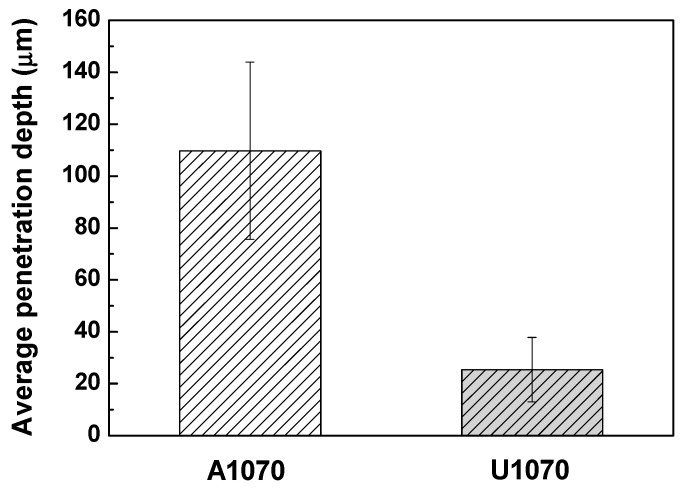
Average corrosion penetration depth of A1070 and U1070 specimens after polarization tests.

Figure 3 shows the polarization curves and schematic Tafel slopes of A1070 and U1070 in a salt water acetic acid solution at 47 °C. Parameters derived from the polarization curve are listed in Table 5. Values from the anodic and cathodic Tafel slopes were similar in both specimens, indicating that the types of cathodic and anodic reactions were not changed. However, the corrosion potential (*E*_corr_) and corrosion current density (*i*_corr_) of U1070 were both lower than those of A1070. As shown in the schematic Tafel slopes in Figure 3b, the shift of the cathodic Tafel slope to the direction of low current resulted in a decrease in *E*_corr_ and *i*_corr_ for U1070. This finding implies that the cathodic reaction intensity was reduced in the case of U1070. The cross-sectional morphologies of both specimens were observed after the polarization tests (Figure 4). As shown in Figure 4, the corrosion rate and the penetration depth were much higher in the case of A1070 than that of U1070. Also, the average corrosion penetration depth was about five times higher for A1070 (109.74 ± 34.19 μm) than U1070 (25.44 ± 12.43 μm), as shown in Figure 5. This means that the actual corrosion behavior was changed due to the Zr alloying. Generally, the cathodic reaction of an Al alloy is primarily related to IMPs, which commonly have a higher potential than that of the Al matrix. Based on the galvanic corrosion mechanism [36], the number of IMPs and the significant difference in the corrosion potential between the IMPs and the Al matrix affect the total corrosion reaction, particularly the cathodic reaction, as IMPs normally act as a cathode. Consequently, the total electrochemical reaction increased due to the high galvanic corrosion condition, which is related to the increase of the number and the size of IMPs. Thus, the corrosion reaction decrease in U1070 could be related to the decrease in the number of IMPs and a difference in the corrosion potential in comparison with the Al matrix. The change of IMPs in both specimens is discussed in the next section, Section 3.2.

**Figure 6:**
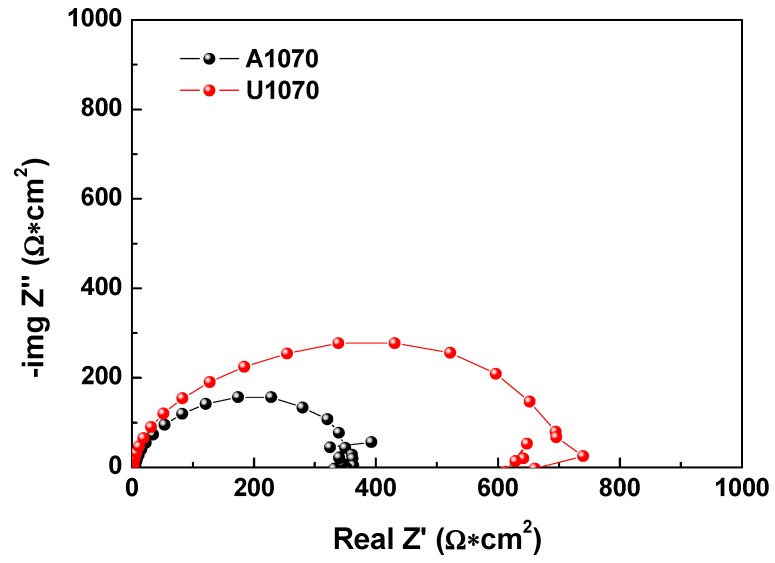
Nyquist plot of A1070 and U 1070 in a salt water–acetic acid solution at 47 °C.

**Figure 7:**
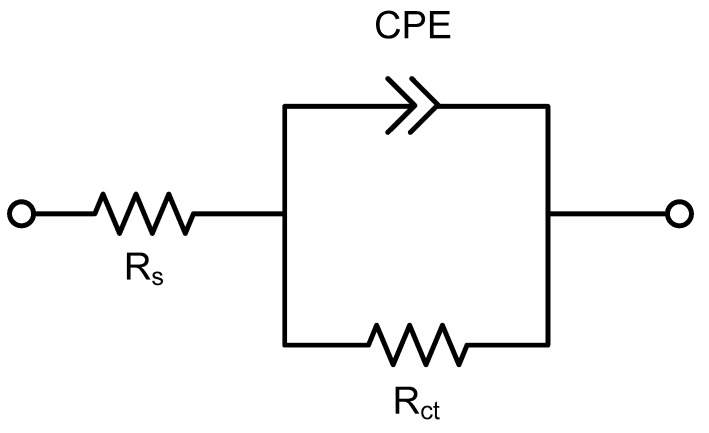
Equivalent circuit for fitting the EIS results for A1070 and U1070 aluminum alloys in a salt water–acetic acid solution at 47 °C.

In Nyquist plots of A1070 and U1070 (Figure 6), the diameter of U1070 was larger than that of A1070, indicating that the addition of Zr increased the corrosion resistance. Depressed semicircles were shown, and capacitive loops, which can be related to the charge transfer reaction and the electrical double layer on the metal surface, were observed [37]. Also, an inductive loop in a low frequency range was observed, which is associated with the weakening of the protective effectiveness of the Al oxide layer due to the anodic dissolution of alloy in a salty environment [38]. Figure 7 presents the electrical equivalent circuit that was used for fitting the EIS spectra wherein *R*_s_ represents the solution resistance, *R*_ct_ represents the charge transfer resistance, and *CPE* represents the constant phase element. In high concentrations of a chloride and acid solution, the Al oxide film does not have passivity, meaning that the effect of the oxide film on corrosion resistance would be neglected. Thus, the simple *R*(*RC*) equivalent circuit was used in this study. The CPE exponent measures the deviation from the idea capacitive behavior [39,40,41]. Table 6 lists the parameters extracted from the EIS data of A1070 and U1070. The double layer capacitance (*C_dl_*) was calculated by use of the following Equation (6) [42]:

(6) $$C_{dl}=Y_{0}{\left(2\pi f_{max}\right)}^{n-1},$$

where *f_max_* is the frequency at which the imaginary component of the impedance reaches the maximum value. The *R*_ct_ of U1070 is higher than that of A1070, indicating that the corrosion resistance of U1070 is higher. These results correlate well with the results of the polarization curve.

### 3.2. Analysis of IMPs

**Figure 8:**
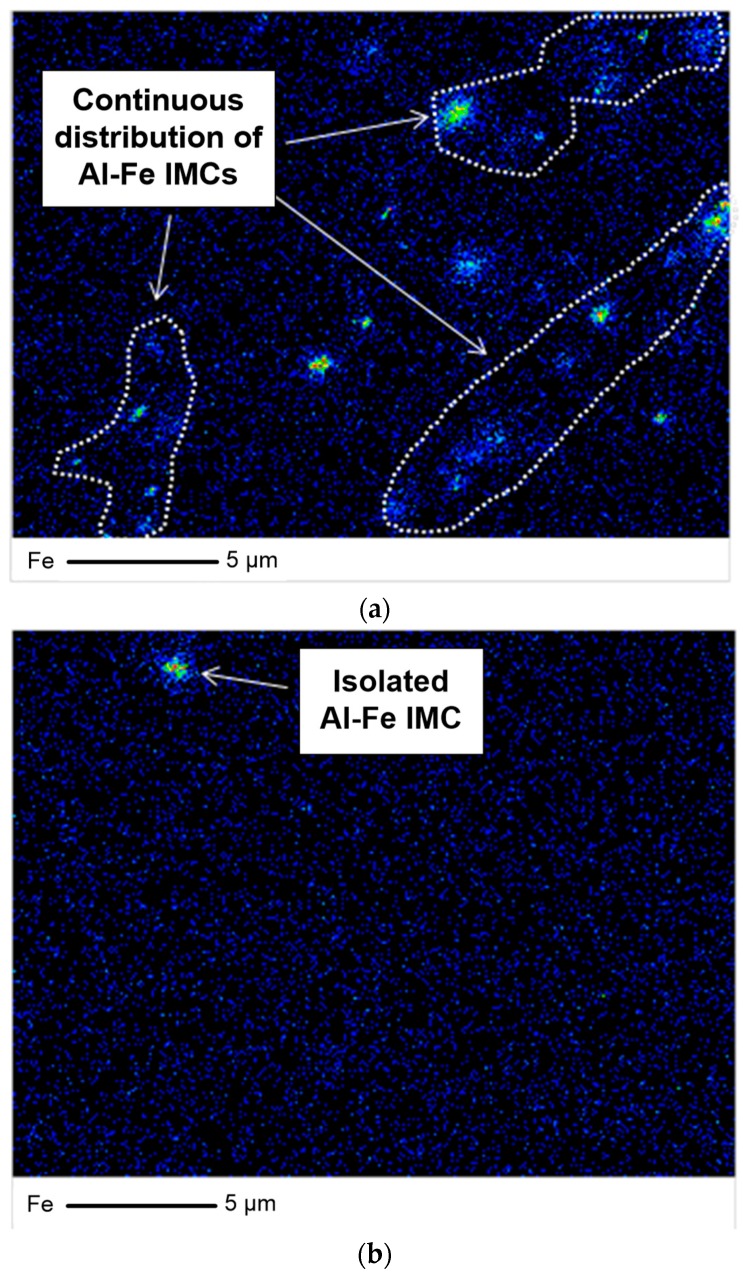
Analysis of Al-Fe IMP distribution using field emission electron probe microanalysis (FE-EPMA) mapping: (**a**) A1070 and (**b**) U1070.

Figure 8 shows the FE-EPMA images of A1070 and U1070 for the analysis of Al–Fe IMP distribution. More Al–Fe IMPs were observed, and it was continuously distributed in the case of A1070 compared with U1070. This finding indicates that the addition of Zr influences the distribution of Al–Fe IMPs. Although there were Al–Fe IMPs in U1070, the total ratio of Al–Fe IMPs would be similar in both Al alloys, because the same amount of Fe was added in the casting process. Thus, it implies that finer IMPs would be formed in the U1070; so a more nanoscale analysis was conducted.

**Figure 9:**
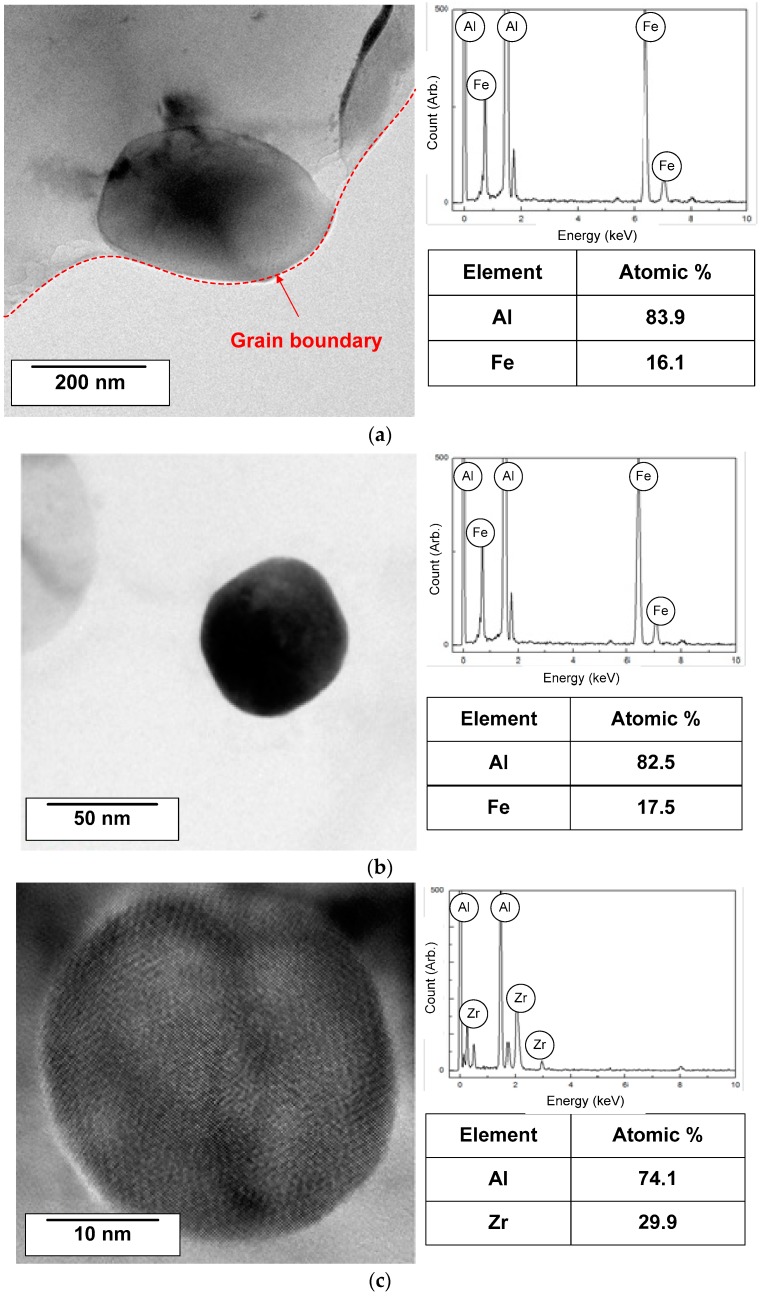
FE-TEM images and chemical composition of (**a**) Al-Fe IMP in A1070; (**b**) Al-Fe and (**c**) Al-Zr IMPs in U1070.

**Figure 10:**
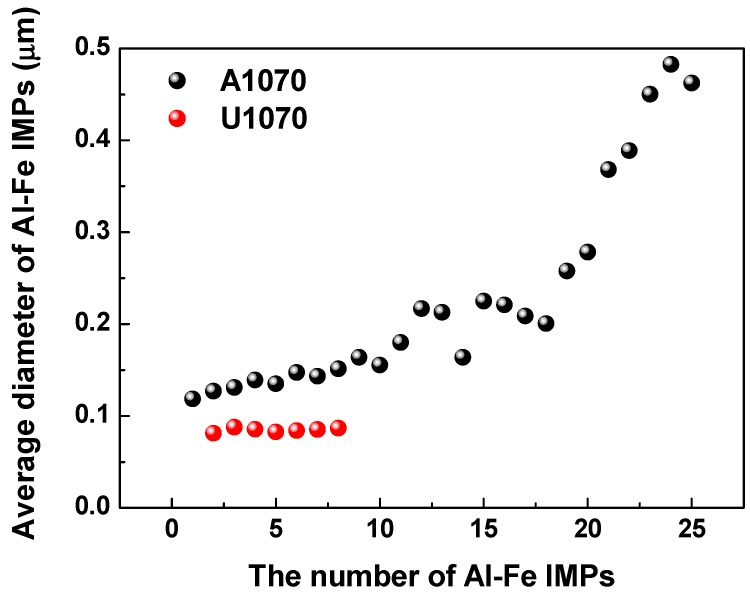
Size and number of Al–Fe IMPs at 300 μm^2^ from FE-EPMA and FE-TEM analyzes.

FE-TEM images of Al–Fe IMPs in A1070 as well as Al–Fe and Al–Zr IMPs in U1070 are shown in Figure 9. In the case of A1070 (Figure 7a), Al-Fe IMPs were formed at the grain boundary, and their diameter of was approximately 250 nm. Meanwhile, Al–Fe and Al–Zr IMPs were not observed at the grain boundary, but rather existed inside the grain in the case of U1070. In addition, the diameters of IMPs (25 nm) for both in U1070 were much smaller than those in A1070. This indicates that the growth of IMPs is depressed in U1070, which may be due to Zr alloying on the refinement of IMPs. In previous studies [43,44,45,46], Zr alloying suppresses the recrystallization of aluminum, refines the grains, and improves the mechanical properties. It indicates that Zr inhibits the growth and promotes the nucleation of other intermetallics during the casting process. Also, the size and number of Al-Fe IMPs were evaluated at 300 μm^2^ in both specimens (Figure 10), which clearly shows the different quantity of Al-Fe IMPs due to the addition of Zr. Consequently, the addition of Zr can result in the refining and dispersing of the detrimental IMPs, which would diminish the effect of the IMPs on the corrosion of Al. As a summary of the IMP analysis, a smaller size and decreasing the number of IMPs (Al–Fe and Al–Zr) were shown in U1070. The detailed analysis of the influence of refinement of IMPs on the corrosion estimate of Al was necessary. Thus, the influence of the number and size of the IMPs on the corrosion resistance was indirectly investigated by case studies of numerical corrosion simulation in this study.

### 3.3. Case Study of Numerical Corrosion Simulation

**Figure 11:**
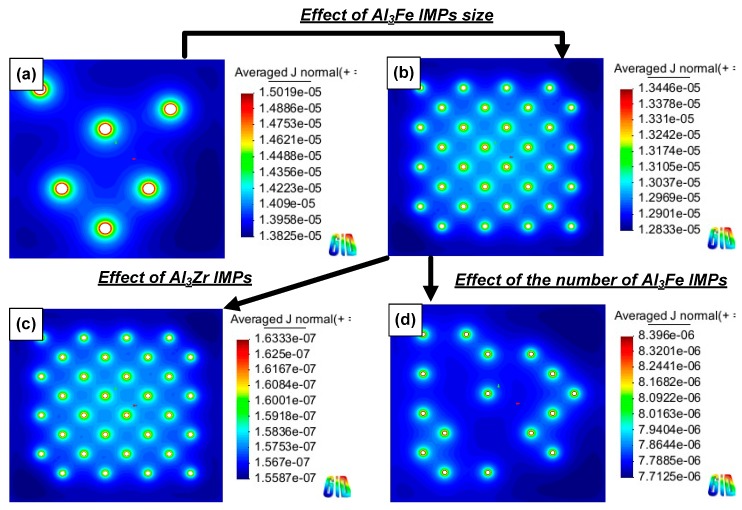
Current distribution of each simulation case; (**a**) large size of IMPs (large IMPs area)–Al_3_Fe; (**b**) small size of IMPs (large IMPs area)–Al_3_Fe; (**c**) small size of IMPs (large IMPs area)–Al_3_Zr; and (**d**) small size of IMPs (small IMPs area)–Al_3_Fe.

Figure 11 shows the current distribution for each simulation case listed in Table 4. In all of the corrosion simulation cases, the current density was high near the Al matrix and IMPs interface, and attenuation of the current density from the interface was observed. This finding indicates that the Al matrix and IMPs act as an anode and cathode, respectively, due to the potential difference. However, the range of current density differs depending on the size, distribution, and type of IMPs that are present. For decreasing Al_3_Fe IMP size (Figure 11a,b), the maximum current density decreased despite similar Al matrix and IMPs areas. This indicates that decreasing the IMP’s size also decreases the maximum current density that is caused by galvanic corrosion between the IMPs and the Al matrix. It may also be related to the localized corrosion behavior of the Al alloy.

In addition, decreasing the number of Al_3_Fe IMPs influenced the current density of the Al matrix, as shown in Figure 11b,d. Although the same Al_3_Fe IMPs acted as a cathode in the simulation, the maximum current density value was decreased, indicating that the galvanic effect between the Al_3_Fe IMPs and the Al matrix was diminished. This occurrence may be due to the IMPs’ interparticle distance. For Al_3_Fe IMPs having a large IMP area (Figure 11b), the galvanic effect can be more concentrated near the interface, as compared to a case with a decreasing number of Al_3_Fe IMPs (Figure 11d). This occurs due to the increasing area ratio (cathode/anode), as well as a decrease in the distance between the IMPs and the Al matrix. As a result, more current would be concentrated on the interface area between the IMPs and Al matrix.

For the Al_3_Zr IMPs (Figure 11c), the maximum current density was approximately 10 times lower than that of the Al_3_Fe IMPs. This result indicates that galvanic corrosion within the Al matrix is insignificant compared to the Al_3_Fe IMPs. In other words, the formation of Al_3_Zr IMPs is not an important factor in the galvanic corrosion of an Al alloy. Consequently, the sum of current from the Al matrix was decreased according to the following situations: (1) the small size of IMPs; (2) decreasing the number of IMPs; and (3) a lower difference in the corrosion potential between the Al matrix and the IMPs.

**Figure 12:**
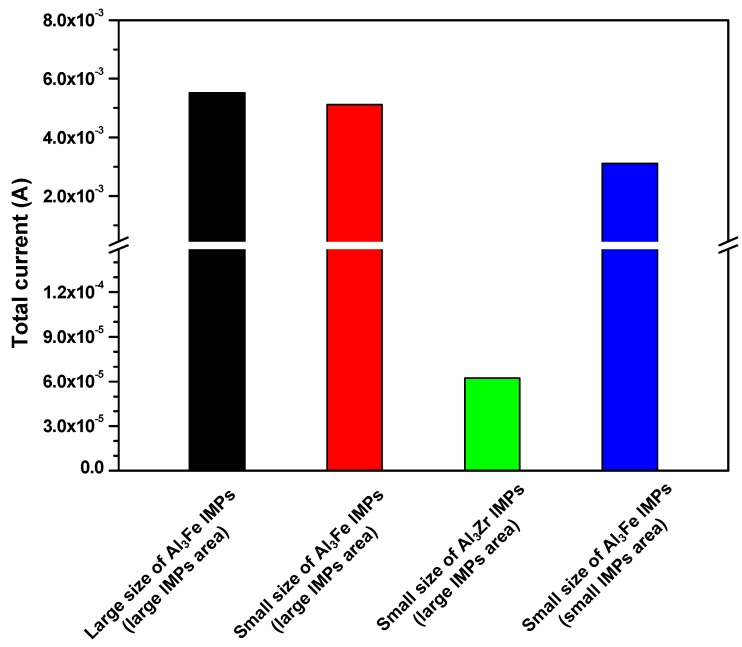
Total current from the Al matrix in each case of corrosion simulations.

The above was confirmed by measurement of the total current from the Al matrix in each simulation case (Figure 12). Total current, which is related to the corrosion current density, decreased in the cases of finely distributed small size IMPs. Also, the total current for the Al_3_Zr IMPs was decreased. These findings regarding corrosion simulation imply that the corrosion current density decrease in Section 3.1 is the result of decreasing the size and the number of IMPs. Also, it was revealed that the Al_3_Zr IMPs do not affect the galvanic corrosion of the Al alloy. Thus, the addition of Zr to an Al alloy is beneficial for improving corrosion resistance, as it reduces the size and number of the IMPs.

## 4. Conclusions

The Zr effect on the corrosion behavior of an Al alloy (1xxx series) was investigated in this study by the potentiodynamic polarization test and EIS. Additionally, FE-EPMA and FE-TEM were conducted to determine the effect of IMPs on an Al matrix, followed by numerical corrosion simulation to investigate the influence of IMPs as a function of distribution and size. In the corrosion tests, the addition of Zr to an Al alloy (U1070) increased the corrosion resistance, indicating that Zr has a beneficial effect on the corrosion resistance of the Al alloy. From the FE-EPMA and FE-TEM analyses, the size of Al_3_Fe IMPs was found to be decreased, and a decreasing number of IMPs were observed in U1070. A case study of numerical corrosion simulation revealed that decreasing the number and size of IMPs is favorable for reducing the galvanic corrosion between the IMPs and the Al matrix. Also, the effect of Al_3_Zr IMPs on the galvanic corrosion of the Al alloy is insignificant as a result of the reduced difference in the corrosion potential between the IMPs and the Al matrix. In summary, the addition of Zr to an Al alloy has a very positive effect in terms of the corrosion resistance, because it decreased the galvanic corrosion effect between the IMPs and the Al matrix by eliciting a decreasing number and size of IMPs.

## Figures and Tables

**Table 1:** Chemical compositions of A1070 and U1070 aluminum alloys (wt %).

Materials	Fe	Cu	Si	Zr	Al
A1070	0.1104	0.0046	0.1519	-	Rem.
U1070	0.1087	0.0037	0.1498	0.1991	Rem.

**Table 2:** Chemical composition of the salt water–acetic acid solution.

pH	Acetic Acid	NaCl
2.9	20 mg/L	4.2 wt %

**Table 3:** Polarization parameters of IMPs and the Al matrix used for corrosion simulation.

Boundary	Corrosion Potential (mV_SCE_)	Corrosion Current Density (A/cm^2^)	Tafel Slope (V/Decade)
Al matrix	−823	3.9 × 10^−6^	0.1
Al_3_Fe	−539	2.1 × 10^−6^	−0.7
Al_3_Zr	−776	2.5 × 10^−6^	−0.7

**Table 4:** Conditions of each IMP’s distributed model.

Simulation Case	Model	Diameter of IMP (mm)	Area of IMPs (mm^2^)	Type of IMPs
1	Coarse IMPs (large area)	0.12	0.0678	Al_3_Fe
2	Fine IMPs (large area)	0.02	0.0678	Al_3_Fe
3		0.02	0.0678	Al_3_Zr
4	Fine IMPs (small area)	0.02	0.0339	Al_3_Fe

**Table 5:** Parameters extracted from the polarization curves of A1070 and U1070.

Specimen	*β*_a_ (V/Decade)	*β*_c_ (V/Decade)	*i*_corr_ (μA/cm^2^)	*E*_corr_ (V_SCE_)
A1070	0.072 ± 0.005	0.123 ± 0.01	98.6 ± 2.5	−0.845 ± 0.05
U1070	0.089 ± 0.003	0.137 ± 0.01	31.5 ± 1.9	−0.911 ± 0.09

**Table 6:** Parameters extracted from electrochemical impedance spectroscopy (EIS) data of A1070 and U1070.

Specimen	*R*_s_ (Ω × cm^2^)	*R*_ct_ (Ω × cm^2^)	*n*	*C*_dl_ (μF/cm^2^)
A1070	4.77 ± 0.5	357 ± 11	0.98 ± 0.02	81.5 ± 5
U1070	1.98 ± 0.3	683 ± 35	0.95 ± 0.03	116.4 ± 9

## References

[1] Davis J.R. (1999). Corrosion of Aluminum and Aluminum Alloys.

[2] Akers W.W., Deans H.A., Crosser O.K. (1959). Condensation Heat Transfer within Horizontal Tubes. Chem. Eng. Prog..

[3] Akersm W.W., Rosson H.F. (1960). Condensation inside a horizontal tube. Chem. Eng. Prog. Symp. Ser..

[4] Cavallini A., Del Col D., Doretti L., Matkovic M., Rosseto L., Zilio C. (2005). Condensation heat transfer and pressure gradient inside multiple minichannels. Heat Transf. Eng..

[5] Adams T.M., Dowling M.F., Abdel-Khalik S.I., Jeter S.M. (1999). Applicability of traditional turbulent single phase forced convention correlations to non-circular microchannels. J. Heat Mass Transf..

[6] Abdulstaar M.A., Mhaede M., Wagner L. (2014). Corrosion behaviour of Al 1050 severely deformed by rotary swaging. Mater. Des..

[7] Chen G., Chen Q., Wang B., Du Z. (2015). Microstructure evolution and tensile mechanical properties of thixoformed high performance. Met. Mater. Int..

[8] Davoodi A., Pan J., Leygraf C., Norgren S. (2005). In situ investigation of localized corrosion of aluminum alloys in chloride solution using integrated EC-AFM/SECM techniques. Electrochem. Solid-State Lett..

[9] Park J.O., Paik C.H., Huang Y.H., Alkire R.C. (1999). Influence of the Fe-Rich intermetallic inc1usions on pit initiation on aluminium alloys in aerated NaCl. J. Electrochem. Soc..

[10] Rynders R.M., Paik C.H., Ke R., Alkire R.C. (1994). Use of in situ atomic force microscopy to image corrosion at inclusions. J. Electrochem. Soc..

[11] Campestrini P., Westing E.P.M., Rooijen H.W., Wit J.H.W. (2000). Relation between microstructural aspects of AA2024 and its corrosion behaviour investigated using AFM scanning potential technique. Corros. Sci..

[12] Ilevbare G.O., Schneider O., Kelly R.G., Scully J.R. (2004). In situ laser scanning microscopy of AA 2024-T3 corrosion metrology: I. localised corrosion of particles. J. Electrochem. Soc..

[13] Birbilis N., Cavanaugh M.K., Buchheit R.G. (2006). Electrochemical behavior and localized corrosion associated with Al_7_Cu_2_Fe particles in aluminum alloy 7075-T651. Corros. Sci..

[14] Davoodi A., Pan J., Leygraf C., Norgren S. (2008). The role of intermetallic particles in localized corrosion of an aluminum alloy studies by SKPFM and integrated AFM/SECM. J. Electrochem. Soc..

[15] Lacroix L., Ressier L., Blanc C., Mankowski G. (2008). Combination of AFM, SKPFM, and SIMS to study the corrosion behavior of S-phase particles in AA2024-T351. J. Elecrochem. Soc..

[16] Bong A., Taylor R.J., Muster T.H., Goodman N., McCulloch C., Ryan C., Rout B., Jamieson D., Hughes A.E. (2010). Stable pit formation on AA2024-T3 in a NaCl environment. Corros. Sci..

[17] Yin L., Jin Y., Leygraf C., Birbilis N., Pan J. (2017). Numerical simulation of micro-galvanic corrosion in Al alloys: Effect of geometric factors. J. Electrochem. Soc..

[18] Shabestari S.G. (2004). The effect of iron and manganese on the formation of intermetallic compounds in aluminum-silicon alloy. Mater. Sci. Eng. A.

[19] Chen C., Wang J., Shu D., Li P., Xue J., Sun B. (2011). A novel method to remove iron impurity from aluminium. Mater. Trans..

[20] Taghaddos E., Hejazi M.M., Taghiabadi R., Shabestari S.G. (2009). Effect of iron-intermetallics on the fluidity of 413 aluminum alloy. J. Alloys Compd..

[21] Kim P., Hayashi C., Nakashima K., Yamada Y., Tachikawa K., Mitsugashira T. (2002). Some characteristics of highly purified aluminium. Phys. Status Solidi.

[22] Samuel F.H., Samuel A.M., Doty H.W. (1996). Factors controlling the type and morphology of Cu-containing phases in 319 Al alloy. AFS Trans..

[23] Flores A., Sukiennik M., Castillejos A.H., Acosta F.A., Escobedo J.C. (1998). A kinetic study on the nucleation and growth of the Al_8_FeMnSi_2_ intermetallic compound for aluminum scrap purification. Intermetallics.

[24] Cao X., Campbell J. (2004). The solidification characteristics of Fe-rich intermetallics in Al-11.5Si-0.4Mg Cast alloys. Met. Mater. Trans..

[25] Davignon G., Serneels A., Verlinden B., Delaey L. (1996). An isothermal section at 550 °C in the Al-rich corner of the Al-Fe-Mn-Si system. Metall. Trans. A.

[26] Moraes H.L., Oliveira J.R., Espinosa D.C.R., Tenorio J.A.S. (2006). Removal of iron from molten recycled aluminum through intermediate phase filtration. Mater. Trans..

[27] Hosseinifar M., Malakhov D.V. (2008). Effect of Ce and La on microstructure and properties of a 6xxx series type aluminum alloy. J. Mater. Sci..

[28] Shabestrai S.G., Mahmudi M., Emamy M., Campbell J. (2002). Effect of Mn and Sr on intermetallics in Fe-rich eutectic Al-Si alloy. Int. J. Cast Met. Res..

[29] Gao J.W., Shu D., Wang J., Sun B.D. (2007). Effects of Na_2_Ba_4_O_7_ on the elimination of iron from aluminum melt. Scr. Mater..

[30] Hashimoto E., Ueda Y. (1994). Zone refining of high-purity aluminium. Mater. Trans..

[31] Crossley F.A., Mondolfo L.F. (1951). Mechanism of grain refinement in aluminum alloys. JOM.

[32] Wang F., Qiu D., Liu Z.-L., Taylor J.A., Easton M.A., Zhang M.-X. (2013). The grain refinement mechanism of cast aluminium by zirconium. Acta Mater..

[33] (2011). Standard Practice for Modified Salt Spray (Fog) Testing, ASTM G85.

[34] Adey R.A., Niku S.M. (1998). Computer modelling of galvanic corrosion. ASTM Spec. Tech. Publ..

[35] Birbilis N., Buchheit R.G. (2005). Electrochemical characteristics of intermetallic phases in aluminum alloys. J. Electrochem. Soc..

[36] Jones D.A. (1995). Principle and Prevention of Corrosion.

[37] Ahamad I., Prasad R., Quraishi M.A. (2010). Thermodynamic, electrochemical and quantum chemical investigation of some Schiff bases as corrosion inhibitors for mild steel in hydrochloric acid solutions. Corros. Sci..

[38] Keddam M., Kuntz C., Takenouti H., Schustert D., Zuili D. (1997). Exfoliation corrosion of aluminium alloys examined by electrode impedance. Electrochim. Acta.

[39] Kim K.H., Lee S.H., Nam N.D., Kim J.G. (2011). Effect of cobalt on the corrosion resistance of low alloy steel in sulphuric acid solution. Corros. Sci..

[40] Lopez D.A., Simison S.N., Sanchez S.R. (2003). The influence of steel microstructure on CO_2_ corrosion. EIS studies on the inhibition efficiency of benzimidazole. Electrochim. Acta.

[41] Bentiss F., Lebrini M., Vezin H., Chai F., Traisnel M., Lagrene M. (2009). Enhanced corrosion resistance of carbon steel in normal sulphuric acid medium by some macrocyclic polyether compounds containing a 1,3,4-thiadizole moiety: AC impedance and computational studies. Corros. Sci..

[42] Hsu C.H., Mansfeld F. (2001). Technical note: Concerning the conversion of the constant phase element parameter Y_0_ into a capacitance. Corrosion.

[43] Tsivoulas D., Robson J.D. (2015). Heterogeneous Zr solute segregation and Al_3_Zr dispersoid distributions in Al-Cu-Li alloys. Acta Mater..

[44] Tsivoulas D., Robson J.D., Sigli C., Prangnell P.B. (2012). Interaction between zirconium and manganese dispersoid-forming elements on their combined addition in Al-Cu-Li alloys. Acta Mater..

[45] Jia Z., Hu G., Forbod B., Solberg J.K. (2007). Effect of homogenization and alloying elements on recrystallization resistance of Al-Zr-Mn alloys. Mater. Sci. Eng. A.

[46] Knipling K.E., Karnesky R.A., Lee C.P., Dunand D.C., Seidman D.N. (2010). Precipitation evolution in Al-0.1Sc, Al-0.1Zr and Al-0.1Sc-0.1Zr (at.%) alloys during isochronal aging. Acta Mater..

